# Midwives’ experiences of performing maternal observations and escalating concerns: a focus group study

**DOI:** 10.1186/s12884-017-1472-8

**Published:** 2017-09-02

**Authors:** Justine Jeffery, Alistair Hewison, Laura Goodwin, Sara Kenyon

**Affiliations:** 1 Heart of England NHS Foundation Trust, Bordesley Green East, Birmingham, B9 5SS UK; 2 School of Nursing, Institute of Clinical Sciences, University of Birmingham, Birmingham, B15 2TT UK; 3 Public Health Institute of Applied Health Research, University of Birmingham, Birmingham, B15 2TT UK

**Keywords:** Midwifery, Maternal observations, Escalation, Safety, Maternal deaths, Barriers, Facilitators, Early warning scoring systems

## Abstract

**Background:**

For the past decade, Maternal Mortality Reports, published in the United Kingdom every three years, have consistently raised concerns about maternal observations in maternity care. The reports identify that observations are not being done, not being completed fully, are not recorded on Early Warning Score systems, and/or are not escalated appropriately. This has resulted in delays in referral, intervention and increases the risk of maternal morbidity or mortality. However there has been little exploration of the possible reasons for non-completion of maternal observations.

**Methods:**

The aim of this study was to explore midwives’ experiences of performing maternal observations and escalating concerns in rural and urban maternity settings in the West Midlands of England. A qualitative design involving a series of six focus groups with midwives and Supervisors of Midwives was employed to investigate the facilitators of, and barriers to the completion of maternal observations.

**Results:**

Eighteen Midwives and 8 Supervisors of Midwives participated in a total of 6 focus groups. Three key themes emerged from the data: (1) Organisation of Maternal Observations (including delegation of tasks to Midwifery Support Workers, variation in their training, the care model used e.g. one to one care, and staffing issues); (2) Prioritisation of Maternal Observations (including the role of professional judgement and concerns expressed by midwives that they did not feel equipped to care for women with complex clinical needs; and (3) Negotiated Escalation (including the inappropriate response from senior staff to use of Modified Early Warning Score systems, and the emotional impact of escalation).

**Conclusions:**

A number of organisational and cultural barriers exist to the completion of maternal observations and the escalation of concerns. In order to address these the following actions are recommended: standardised training for Midwifery Support Workers, review of training of midwives to ensure it addresses the increasing complexity of the maternal population, identification and agreement regarding the organisation of maternal observations among staff, an emphasis on increasing the priority placed on maternal observations in all clinical settings, and clarification and reinforcement of escalation procedures for both midwives and senior clinicians.

**Electronic supplementary material:**

The online version of this article (10.1186/s12884-017-1472-8) contains supplementary material, which is available to authorized users.

## Background

In maternity care, the completion of maternal observations refers to the measurement of women’s temperature, heart rate, respiratory rate and blood pressure, during the antenatal, intrapartum and postnatal period of maternity care [1–3]. In the United Kingdom (UK), the standards for the frequency of undertaking and recording these observations are set by the National Institute for Health and Care Excellence (NICE) [1–3]. NICE is an executive non-departmental public body of the Department of Health in the United Kingdom responsible for the provision of national guidance and advice to improve health and social care. National and local guidelines also specify the action to be taken to escalate concerns if such observations are outside of the normal range – for example informing the coordinating midwife and obstetrician, or transferring women to obstetric-led care [3].

However statements of concern about the completion of maternal observations have been a recurring theme in the Confidential Enquiry into Maternal Deaths (CEMD) reports for over a decade [4, 5]. It is noted that maternal observations are sometimes not undertaken, not undertaken completely, and/or not recorded on early warning scoring charts, and abnormal observations are not escalated appropriately to senior clinicians [4, 5]. The failure to complete observations and escalate action results in delays in intervention and increases the risk of maternal morbidity and has been a contributory factor in a number of avoidable deaths [4, 5]. In 2007, and again in 2011, the CEMD recommended the introduction of a Modified Early Warning Scoring System (MEWS) in the UK to increase the completion rate of maternal observations, enable the early identification of deterioration, and improve communication at the time of escalating concerns [5, 6]. MEWS charts are used to calculate ‘scores’ for women’s heart rate, respiratory rate, blood pressure, consciousness and temperature, which are then added together to create an overall MEWS score which indicates any deterioration in condition [6]. The rationale for the use of MEWS charts is that recording a combination of small changes in these five variables will enable detection of deterioration in condition earlier than waiting for a major change in one observation such as reduced blood pressure or raised temperature before taking action. In this way timely recognition, treatment and referral of women who have, or are developing, a critical illness is enabled [6]. Although the uptake of the use of MEWS charts in obstetric units in the UK is reported to be 99% [7], the CEMD continues to identify problems with the completion and reporting of maternal observations in maternity care [4, 6, 8].

Previously, the identification of an area of clinical concern in a CEMD report has resulted in action being taken to address the particular issue. For example in response to the findings that thrombosis and sepsis had contributed to maternal deaths the Royal College of Obstetricians and Gynaecologists (RCOG) produced the guidelines for *‘Thromboprophylaxis during Pregnancy, Labour and after Vaginal Delivery’* [9], and the guideline on *‘Sepsis in Pregnancy and Sepsis following Pregnancy’* [10]. While no formal evaluation of the impact of these guidelines was undertaken, the reductions in incidence of thrombosis and sepsis noted in subsequent CEMD reports [4, 5], suggest they have had some influence on clinical practice. Yet, the recommendations made for the completion of maternal observations have not resulted in any evidence of improvement in their completion or reduction in mortality [4, 5]. This is of particular concern in view of the complex nature of the health problems experienced by an increasing number of women which need to be managed alongside the pregnancy [11–14]. Moreover the age at which mothers in the UK give birth is increasing and there are fewer births to younger women and more to older women [15], which adds to the challenge of caring for mothers and highlights the importance of clinical observations as part of maternity care. For example, in 2015 England had over 70,000 more births to women in their thirties than in 2001, and for women aged 40 or older, there were over 12,000 more births [15]. The current national shortage of midwives in the UK [15] places additional pressure on maternity services, further increasing the need for problems with the completion and reporting of maternal observations to be addressed.

Midwifery supervision also appears to have had little effect on midwives’ undertaking of maternal observations. Supervisors of Midwives (SoMs) were introduced in 1902 [16] to improve midwives’ standards of care by identifying and investigating sub-standard practice and making recommendations to address concerns [17]. As such, the SoM role encompasses monitoring standards of clinical practice, including the completion of maternal observations [5, 6]. However it is not clear if the role has had any discernible impact on the completion of maternal observations. Furthermore, the statutory function of SoMs was deregulated by the Department of Health in March 2017 following criticism of their role as a flawed because it was an ineffective “additional layer of regulation” [18] between the service and the health care professional regulator, the Nursing and Midwifery Council (NMC). Responsibility for monitoring standards of clinical care now rests solely with the employer [19].

Similarly, changes to the content of pre-registration midwifery training, introduced by the NMC [20] appear to have had little effect. For example, whilst maternal observations are now included in the ‘normal labour and birth’ section of the pre-registration standards [20], the guidance lacks clarity as it does not specify all of the maternal observations required to make a full clinical assessment, and can be found only in the cluster relating to intrapartum care. There is no specific reference to temperature, pulse, respiratory rate or blood pressure in the antenatal or postnatal skill clusters [20].

### The literature

A systematic search of the midwifery literature identified only three publications exploring the completion of maternal observations [7, 21, 22]. In view of this, nursing literature was accessed, and the search terms expanded to include clinical observations of adults in nursing. This returned a further 12 publications. Despite the lack of midwifery literature, there were some common findings reported in both the nursing and midwifery literature concerning barriers and facilitators to the completion of clinical observations. The lack of staff was one of the most frequently reported barriers preventing the completion of observations [7, 21, 23–26]. Indeed, a number of nursing studies found that completion rates fell at night, weekends, and on public holidays, because there were fewer senior staff and/or more agency staff on duty [7, 21, 27]. One study also reported a correlation between ‘out of hours’ periods (including night time) and non-completion of observations [24], which was attributed to reduced staffing during these times [24]. It was also reported in the nursing literature that reductions in qualified staff resulted in an increased reliance on support staff to complete clinical tasks, including observations [23, 26]. This hindered their completion because support staff did not always have the appropriate training or experience necessary to complete observations in accordance with the correct procedures [23, 25]. There were no reports of support staff undertaking maternal observations in the midwifery literature.

In the nursing literature, negative attitudes towards observations on the part of support staff were also implicated in contributing to non-completion [24, 26]. For example, unregistered staff reported that the completion of observations is ritualistic, time-consuming, of low priority and required little clinical skill, and so they are often not undertaken [24, 26]. It has also been suggested that reliance on support staff can delay the escalation of concerns, particularly in situations where they are unable to identify deterioration and are uncertain about their responsibilities with regard to escalation [25].

In the midwifery literature, clinical location was identified as a factor affecting the completion of maternal observations [21, 22]. For example, women being cared for in areas with 1:1 midwifery care, such as delivery suite or high dependency units, were more likely to have observations completed at the prescribed intervals than women in midwife-led units and those being cared for in the community [21]. The lack of a nationally agreed Early Warning System (EWS) in obstetrics was also identified as a barrier to the completion of maternal observations and escalation of concerns [7, 21]. Early warning systems are used in hospitals to ‘track’ a patient’s condition, to enable early detection of deterioration and ‘trigger’ appropriate clinical intervention. However, maternal physiology is different from the non-pregnant state therefore the parameters for deterioration and escalation differ from those included in the general EWS charts [21]. Despite recommendations for standardised EWS charts nationally [5, 6, 28], there is variation in application in different Trusts, and even differences in use among units in the same Trust [21]. Consequently, uncertainty about when to escalate concerns is increased by the variation in how the recordings of observations are interpreted in different settings and can therefore act as a barrier to reporting abnormal scores [21].

In the wider discourse of UK midwifery, emphasis is placed on the ‘normality’ of birth [29], and labouring women are therefore viewed as experiencing a ‘normal event’ [29]. This is reflected in the midwifery literature where the purpose of regular clinical observations in midwifery practice is questioned. Mackintosh et al. [22] found that midwives felt clinical observations were unnecessary and not consistent with the view of birth as a ‘natural’ process. Instead midwives used their clinical judgement, gut feelings and intuition to decide if women needed observations [22] and prioritised breast feeding support, observations of the baby, and managing discharge processes for women seen as ‘normal’ [22]. This reliance on professional judgement in preference to clinical observation has also been described by Isaacs et al. [21], who found that practitioners relied on their experience to make decisions about care, rather than completing maternal observations as directed by procedures. In the nursing literature there was some evidence of non-compliance extending to falsification of recordings noted on observation charts [30]. This behaviour was explained as a way to avoid the need to trigger the escalation process if the nurses felt the patient did not need to be reviewed by senior staff [30].

Barriers to escalating concerns were also identified in both the nursing and midwifery literature [7, 22, 26, 30–33]. One such barrier was the nature of escalation protocols which were seen to have a negative impact on the timing of escalation, as well as the willingness of staff to escalate in future. Indeed, within the midwifery literature the use of different risk scoring systems and parameters for escalation across sites were seen to reduce staff confidence in the process and decrease the likelihood of them escalating concerns in the future [7, 22]. In the nursing literature, some escalation protocols were also seen to cause delays in the process because of the number of steps involved - requiring the staff member to consult a unit manager before contacting medical staff for example [26, 30]. In both the nursing and midwifery literature, inappropriate responses from medical staff were found to be a barrier to future escalation of concerns [7, 31]. The fear of ridicule from colleagues and senior staff, should escalation subsequently be deemed a ‘false alarm’, reduced the likelihood of staff initiating this process [32] and nurses reported delaying escalation until they had discussed their concerns with senior staff to avoid being reprimanded and/or appearing foolish [33]. Along with concerns about appearing foolish staff may also experience stress, anxiety and excitement when initiating escalation procedures [32], suggesting that there is an emotional cost associated with escalation. In both the nursing and midwifery literature, escalation of concerns to senior staff was sometimes regarded as evidence of staff being unable to ‘cope’ or ‘manage’ patient issues themselves [22, 31]. Such assumptions arguably constrain escalation as staff do not want to be perceived as unable to meet the requirements of their role.

Although some literature exists to explain non-completion of maternal observations and escalation of concerns, the findings are drawn from a relatively limited body of nursing and midwifery research, which is of variable quality. Furthermore, few studies have explored healthcare professionals’ perceptions and experiences of performing maternal observations and escalating concerns, and such exploratory work has not been conducted in the midwifery setting.

## Methods

This qualitative study was designed to explore midwives’ and Supervisors of Midwives’ experiences of performing maternal observations and escalating concerns in a maternity care setting. The aim of the study was to investigate the reasons for non-completion of observations and the issues surrounding the escalation of concerns, by accessing the participants’ accounts of their views and experiences. In addition the intention was to explore potential solutions to any problems identified. As little is known about why midwives do or do not complete maternal observations, a non-experimental, qualitative, approach was identified as the most appropriate to address the aim of this study, in order to obtain rich, in-depth data, and the generation of new insights on this phenomenon [34]. Focus group interviews were used to gather qualitative data, as the interaction between participants in a focus group allows researchers to assess the level of agreement and disagreement on a topic in a short period of time [35], and enables participants not only to present their own views, but also to hear the views and experiences of others. Through active discussion of issues, participants reveal more of their own frame of reference [36].

A purposive sampling approach was taken to recruiting participants, in order to obtain views from those with the most information on the topic, in this case the direct day-to-day experience of performing maternal observations in both the hospital and community setting [37, 38]. The identification of this sample offered the opportunity for the collection of rich data [39], and ensured that a variety of perspectives on the issue being explored were obtained [39].

### Recruitment

Eligible participants were those working as qualified Midwives/Supervisor of Midwives in a maternity setting in one of four sites in the West Midlands of England. These four units included a mix of tertiary and district hospitals, in both rural and urban settings. The Local Supervising Authority (LSA) Midwifery Officer and Heads of Midwifery in the West Midlands were contacted to outline the proposed study and to gain their support. Once support was confirmed, the Head of Midwifery from each Trust was asked to identify a senior member of the midwifery team (in most cases the Matron) to assist with the recruitment and planning of the focus groups. Midwives and SoMs who were interested in participating in the research gave their contact details to this senior member of staff, who then passed them on to the researcher (JJ). Participant information sheets were sent electronically to all interested parties by email, with a request to contact the researcher directly if they were still interested in taking part. Consistent with the purposive sampling approach, and the principle of data saturation [40], recruitment continued until no new significant themes emerged from the data.

### Focus group structure

Six focus groups were conducted in four maternity units in NHS Trusts in the West Midlands of England between January and May 2016. Midwives took part in focus groups with other midwives (four separate groups), and the SoMs participated in groups with other SoMs (two separate groups). The intention was that each focus group would involve between four and eight participants as recommended in the methodology literature [35, 41], however because of staff availability group size ranged from three to six individuals for both midwives and Supervisors of Midwives. Although the groups were smaller than planned this did not appear to have an adverse effect on the duration of discussion or the amount of data generated.

The Focus groups were convened in meeting rooms in the four maternity units and each session lasted on average one hour. Flexible topic guides (included as Additional files 1 and 2) were used to provide a format for the discussion, and included open-ended questions drawn from the literature. The discussions explored the participants’ views and experiences of conducting maternal observations and escalating concerns in maternity settings.

### Analysis

The focus groups were audio-recorded, and an experienced second moderator completed contemporaneous field notes of non-verbal communication including eye contact, gestures, and facial expressions. Audio recordings were then transcribed verbatim by the researcher (including pauses, laughter and prolonged silences), and augmented by the field notes.

The Framework Method [42] was adopted for the management and analysis of the data. This approach began with anonymisation of and familiarisation with the data, followed by the application of a priori *codes* (identified following the review of the existing evidence) to three transcripts. The process of ‘open coding’ was then applied to the same three transcripts to facilitate inductive analysis to identify new and emergent themes in the data. Data of interest were highlighted in the text and assigned a coding label [34]. Coding labels were grouped to identify codes, and refined after a further transcript review [40, 43]. Similar labels were combined and grouped into themes to develop the final Analytical Framework. The analytical framework and a priori codes were applied to the remaining three transcripts and then reviewed. A framework matrix was created in Microsoft Excel, and data were charted into this matrix for interpretation. At each stage of the process emerging codes and themes were discussed, reviewed, refined and agreed with authors AH and SK.

## Results

A total of 18 midwives and 8 Supervisors of Midwives took part in focus groups between January and May 2016. The majority of participants were aged over 40 years (74%), were of white ethnicity (70%), and had been qualified for more than 10 years (66%). Clinical banding ranged from Band 5 (17%) to Band 7 (35%). Full participant demographics are presented below (Table 1).

**Table 1:** Participant Demographics^a^

Demographic variable		Number (n=)	Number (%)
Age Group	20–29 30–39 40–49 50+	3 3 7 10	13 13 30 44
Ethnic Group	White Black Chinese	16 6 1	70 26 4
Job Title	Midwife Supervisor of Midwives	15 8	65 35
Clinical Banding	Band 5 Band 6 Band 7 Other	4 5 8 6	17 22 35 26
Highest Qualification	Diploma Degree Masters/PhD Registered Midwife	1 16 3 3	4 70 13 13
No. of years worked as a midwife	0–1 1–5 6–10 11–15 Over 15	2 5 1 2 13	9 21 4 9 57
Clinical Area	Community Delivery Postnatal Other	2 4 6 11	9 17 26 48

^a^Three participants declined to provide demographic information, leaving 23 participants included in this data set

Three key themes emerged from the data: (1) Organisation of Maternal Observations (including delegation of tasks to Midwifery Support Workers, variation in their training, and the care model used e.g. one to one care and staffing issues); (2) Prioritisation of Maternal Observations (including professional judgement and concerns expressed by midwives that they did not feel equipped to care for women with complex clinical needs); and (3) Negotiated Escalation (including the inappropriate response from senior staff to use of Modified Early Warning Score systems, and the emotional impact of failed escalation).

Data extracts from focus groups with midwives and supervisors of midwives are presented together, as findings were similar. Quotes from midwives are identified as MW, and quotes from supervisors of midwives are identified as SM. Focus groups 1–4 (FG1-FG4) were attended by midwives only, and FG5-FG6 were attended by supervisors of midwives only.

### Organisation of maternal observations

This theme reflected the way maternal observations were organised in maternity units. Delegating tasks to Midwifery Support Workers, the contrasting cultures of different clinical locations, and the effect of staffing and skill mix, were all identified as influencing the organisation of maternal observations. In all but one of the focus groups, midwives reported that clinical observations had been almost completely delegated to the Maternity Support Workers (MSW) other than in areas delivering 1:1 care provided by midwives.

‘*MSW are a great help. They're the ones that do the majority of round observations’ (MW 1, FG 4).*

Indeed, midwives explained that tasks such as making the discharge arrangements for women meant that they were often taken away from the bedside and consequently had to rely on MSWs to complete observations, record the results on a MEWS chart, and report any signs of deviation.

‘*Clinically [midwives] don't necessarily get the chance [to undertake observations], if the ward’s busy and we’re wanting beds, to actually go to the patient*’ *(MW 3, FG 1).*

However, midwives noted inconsistencies in the abilities of the MSWs to identify deterioration and voiced concerns regarding MSWs’ abilities to escalate appropriately. Participants suggested that there was confusion concerning who was responsible for calculating MEWS scores and reporting deterioration, and recounted a number of incidents where MSWs failed to complete these actions:

‘*[MSWs] don't always report back timely if you’ve got an abnormality*’ *(MW 3, FG 1).*

A lack of appropriate training was seen as the cause of the variation in MSW skill levels, and midwives reported that many MSWs were expected to learn how to conduct observations ‘on the job’. Participants reported that the training provided for MSWs was inconsistent across the region, with only one site identifying a clear programme of training for MSWs.

Staffing issues were also reported to have an impact on the way maternal observations were organised. For example to balance the increased workload with reductions in staff numbers, ‘out of hours’ observations were often neglected.

‘*There's only two midwives on the ward at night…when you haven't got a full ward, that's fine, you can cope and you can do everything you need to do. But if you fill up overnight and that ward is full, it’s really difficult to keep on top of everything you need to do’ (MW 3, FG 2).*

Clinical location was also identified as affecting the completion of maternal observations. For example, midwives in two of the focus groups suggested that observations were less likely to be conducted in a freestanding midwifery-led unit or during community sessions than on delivery suite or a high dependency unit. Whilst some midwives suggested that the ratio of women to midwives mediated this relationship:

‘*I mean, the one here (MLU), it's only got three beds, and two midwives, so it's easy to do them, 'cause you're more one on one’ (MW 5, FG 4),*

Others suggested that variation in the completion of observations between clinical locations may be due to the ways in which staffing was organised:

‘*I've had numerous shifts where I'm just on my own (triage). So doing all the obs and all the care and informing the doctors…’ (MW1, FG 3).*

### Prioritising maternal observations

Another key theme to emerge from the data was the range of factors that affected the priority assigned to maternal observations, and consequently influenced whether or not these observations were performed. A combination of factors appeared to confer a lower priority on observations including the concept of normality, high clinical workload, and professional judgement.

The priority given to the completion of maternal observations was also reported to differ between clinical locations – for example between delivery suite and midwifery-led units. It was reported that there were variations in attitude towards clinical measurements between these settings, which affected the priority given to performing these tasks. This was reflected in different guidelines and cultures in each setting:

*‘It’s all low risk care over there [midwifery-led unit], you see, but even so, I mean the amount of times I’ve gone to take over for my postnatal lady and there isn’t even an observation chart, and that happens regularly’ (MW2, FG 2).*

There was agreement amongst midwives that birth was a normal event and should be treated as such, although most of the participants suggested that this view did not negate the need for clinical observations to be performed. However a number of midwives suggested that a focus on normality could contribute to clinical observations being regarded as a low priority:

‘*I think nationally postnatal care and postnatal observations have probably not [been] viewed in the way that they should have been*’ *(SM 3, FG 5).*

Furthermore, many of the junior midwives regarded their normality-focused midwifery training as inadequate preparation for caring for women with complex needs, a view echoed by their more experienced colleagues. Current training programmes were reported to neglect the importance of clinical skills such as maternal observations, which contributed to the lack of their completion in practice. The junior midwives in particular identified differences between themselves and more experienced staff in terms of their ability to identify when women needed increased monitoring in response to pre-existing co-morbidities or developing morbidity.

‘*The older midwives have done nursing, they’re more prepared to deal with an ill woman, whereas us that are coming through new, we’re not used to that, we’re used to normality’ (MW 4, FG 6).*

High clinical workload was also reported to affect the priority given to maternal observations. For example, routine observations were sometimes neglected or delayed in order to maintain continuity of care for women.

*‘…you’re giving clinical care to somebody else, you can’t just say, “I'm stopping now,” and walking away [to do an observation]’ (MW 3, FG 1).*

It was reported that some midwives needed help to prioritise their work in an appropriate way:

*‘It’s helping people think about what’s important and what can wait…And actually a lady that needs breastfeeding support, can that wait for an hour? Get the baby some skin to skin while you go back and check that lady’s not bleeding.’ (MW 3, FG 1).*

Professional judgement was also identified as a potential barrier to prioritising maternal observations in preference to other clinical tasks. Midwives spoke about their autonomy in decision-making, and terms such as ‘gut feelings’ and ‘knowing’ were discussed. Although participants reported that they adhered to the protocols in terms of performing maternal observations, three midwives expressed the view that acting on intuition was more important than recording these observations. Indeed, for women who ‘seemed fine’, a minority of midwives reported the completion of maternal observations to be optional or unnecessary.

### Negotiated escalation

Another issue seen to be central to the completion of observations and escalation of concerns was the use of the Modified Early Warning Scoring System (MEWS), which had been developed locally by multi-professional teams. It was acknowledged that the MEWS charts differed between sites however the midwives generally spoke in positive terms about using them in practice:

*‘I think the MEWS are great, and I think they are fit for purpose’ (MW 3, FG 6).*

What was found to be more challenging though was the variation in response from medical staff when concerns were escalated. The midwives reported that they often had to negotiate with senior staff regarding the urgency of the woman’s condition, rather than action being taken on the basis of the MEWS score, as the system required. For example, midwives gave accounts of senior staff asking them to report which measurement was triggering the MEWS (i.e. identifying precise measurement and type of observation which indicated the woman had abnormal symptoms), often followed by a request to repeat the observations later and call again if the urgency had increased:

*‘Generally, it depends what they're MEWSing on. If it's blood pressure the doctor is supposed to be informed. It's generally just “repeat it in an hour, and see what it is”’ (MW 5, FG 3).*

Consequently, midwives spoke of MEWS charts causing conflict, and using the score as a basis for negotiating escalation with medical staff, rather than using it for its intended purpose as an agreed threshold.

*‘Well, it is doing battle, isn't it? Sometimes getting to the review, you’re having to argue your case’ (MW 2, FG 1).*

On other occasions there was no opportunity for negotiation. For example, accounts were given of medical staff dismissing symptoms which midwives had been trained to recognise as signalling the need for escalation:

*‘I think…sometimes when the MEWS are escalated, some of the doctors can be quite dismissive...so, for example, if you've got a woman who is in labour, and she is in pain, but she's MEWSing, and she's also got pre-eclampsia, they might be dismissive, and [say], “Well, what did you expect, she's in pain?”’ (SM 5, FG 5).*

When this occurred the midwives reappraised symptoms as giving less cause for concern. This had implications for escalation of similar cases in the future because the threshold was recalibrated by the midwives based on the response from medical staff:

*‘So, [the midwife] escalates to a doctor, and because [the doctors] just say, "Oh, that's fine, just repeat in an hour," [midwives] don't tend to think, oh, there's an issue here, because the last time I phoned the doctor, he said it was fine.’ (SM 3, FG 6).*

In three of the focus groups, some junior midwives (band 5) expressed reluctance to contact senior midwifery and medical staff even when abnormal observations had been recorded. They felt this arose from a lack of confidence that they would be taken seriously by senior staff because of their junior status:

*‘Say if you’re either a confident band six, or a band seven, doctors are more likely to probably listen to you. The darker the uniform, the more likely to listen to you’ (MW 5, FG 4).*

In addition to seniority, having a good working relationship with medical staff was felt to ameliorate some of the fears about escalation, and midwives suggested that day-to-day contact with senior staff was a good way to establish these connections. In this way, working on delivery suite was seen to facilitate successful escalation, whilst midwives working on midwifery-led units or in the community, had fewer opportunities to build these important connections:

‘*Working on delivery suite and having more exposure to the consultants [helps escalation], ‘cause our consultants have a high presence, they do rounds. So you do get to know them*’ *(MW 2, FG 4).*

## Discussion

This study was designed to address a gap in the literature by exploring midwives’ experiences of performing maternal observations in a maternity care setting. The novel findings provide a number of helpful insights on this important area of midwifery care. These include the environmental, task-specific, organisational, and professional barriers to performing maternal observations and escalating concerns. The three themes to emerge from the data were the organisation of maternal observations, including training of those undertaking observations (standard training for MSWs and further training for midwives), prioritisation of maternal observations, and negotiated escalation, which are discussed further below.

A key finding of the study was the extent of the delegation of maternal observations to Maternity Support Workers (MSW) by midwives. Although delegation of care tasks to support staff has been reported in the nursing literature for many years [see for examples 23, 25, 26], it has not been noted previously in midwifery care. For the most part, midwives spoke about the delegation of tasks to MSWs in positive terms. Although the lack of awareness of, or adherence to, clinical guidelines, and confusion over whose responsibility it was to complete these observations were identified as areas of concern and noted as barriers to completion. Reliance on support staff to complete maternal observations was also a factor in preventing escalation of concerns. This has been known to be the case in nursing for some years, with healthcare professionals questioning the reliability of support staff in appropriately escalating concerns [25, 26], and criticising the lack of clear leadership and knowledge necessary to identify deterioration from observations [25, 26]. However its significance for midwifery care has not been identified previously. The uncertainty regarding the ability of support workers to record maternal observations and escalate from them appropriately is of concern, given that such tasks are increasingly being delegated to them. Indeed, if MSWs are to complete maternal observations, and deliver high quality and safe clinical care, it is essential that more attention is paid to the level of training and education provided for these staff. The findings from this study also suggest that clarity of roles and responsibilities regarding maternal observations is needed in order to increase completion rates. The need to address this issue is becoming more urgent in light of the national shortage of midwives [15], and the likelihood that reliance on MSWs for of the completion of clinical tasks will increase [15].

When midwives reported carrying out maternal observations themselves, staff shortages were identified as one of the biggest barriers to regular completion. Following the review of the literature, [23, 24] this was an anticipated finding of the study. Indeed, in Isaacs et al.’s study [21], staffing pressures were perceived as the main barrier to the completion of maternal observations, confirming, perhaps unsurprisingly, that staffing levels outside areas with 1:1 staffing ratios need to be addressed if completion of observations is to improve.

The priority given to maternal observations was a key theme in the data, and was influenced by the concept of birth as a normal event. However rather than the concept of normality in and of itself leading midwives to reject the need for observations when caring for ‘normal’ women [22], the midwives suggested that it was the impact of normality-focused training which left ‘newer’ midwives ill prepared to care for women with complex needs on antenatal and postnatal wards, and unsure when routine clinical observations were necessary. This was even more keenly felt in community settings where the overall culture of care was reported to eschew the importance of clinical observations. This suggests that there may be unintended consequences arising from the normalisation, or perhaps more accurately, the de-pathologising of childbirth. This is likely to become a more pressing policy and service delivery issue given the rise in the number of women presenting with multiple and complex illnesses when pregnant, as noted earlier [11–14]. It may be necessary to refocus on clinical observations and measurements in maternity care, to ensure that appropriate priority is placed on maternal observations.

Professional judgement was also identified as factor which influenced the way maternal observations were prioritised. A number of the midwives discussed their reliance on ‘knowing’ and ‘gut feeling’ rather than clinical observations, which was also noted in earlier work [22, 44]. However, other more experienced midwives reported that their professional knowledge was important, but not conclusive, in deciding when abnormal maternal observations should be escalated, consistent with other recent findings [43, 44]. Whilst the majority of midwives in this study reported that professional knowledge did not negate the need for completing and recording maternal observations, the data collection methods utilised by this study make it difficult to interpret whether these are reflections of the behaviours of midwives, or whether the information provided was influenced by concerns about social desirability on the part of the respondents [45]. This caveat aside, the findings from this study and previous literature suggest that the completion and escalation of maternal observations is still influenced in part by professional judgement. As such, steps to standardise and audit the completion of such tasks need to be taken if there is to be an increase in completion rates.

In the current study, negotiation of MEWS scores emerged as the main barrier to escalation of concerns arising from maternal observations. Midwives reported that escalation often led to drawn-out conversations with medical staff, who would request a break-down of MEWS scores. Such conversations frequently ended in midwives being asked to call back if/when women’s observations worsened. This negotiated process of escalation works counter to the purpose of the MEWS as providing an ‘objective’ and agreed threshold for action, and is an important finding. Indeed, rather than issues with MEWS charts themselves (for example inconsistencies in scoring between sites [22]), this study found it is staff responses to escalation that are a barrier to action. Future research may be necessary to explore this in further detail, and to establish whether it is necessary to train staff to report and respond to MEWs scores differently.

Negotiated escalation also had the effect of reducing midwives’ motivation to escalate concerns in the future. For a number of midwives, experiencing unsuccessful, negotiated, or ‘failed’ escalation engendered a fear of anticipated criticism from other staff should further efforts to escalate be made. They felt that future escalation for similar issues might be perceived by other staff as an indication that they could not cope with situations independently and were therefore less skilled or resilient in their role. This echoes findings from nursing in that nurses feared ridicule when escalating patient deterioration [32], and that this fear was a disincentive to completing this action [32]. Along with reviewing current escalation processes, attention should also be given to creating the circumstances in which close working relationships with medical staff can be achieved. It was clear from the focus group discussions that where these relationships were established, escalation was more timely and straightforward. The simple expedient of staff working in a team negated the need for rigid adherence to escalation protocols. One way of addressing this is for midwifery managers to seek ways to establish and maintain these professional relationships – particularly for community staff, for whom one-to-one contact with medical staff is rare.

To the best of our knowledge this is the first study to explore midwives’ experiences of performing maternal observations in a variety of maternity settings in England. It provides useful insights on the barriers and facilitators to the completion of maternal observations in current midwifery practice from the perspective of practising midwives and their supervisors. A strength of this study was its qualitative approach, which allowed for rich and in-depth data to be generated, through rigorous analysis. As no new themes arose in later focus group sessions, data saturation appeared to have been reached during collection, suggesting that the sample size was appropriate and suitable for meeting the research aims [40].

While every effort has been made to make this study as robust as possible, there are limitations which must be acknowledged. For example, many of the barriers to performing maternal observations reported by the participants focused on the roles of other team members and organisational issues rather than their own practice. The presence of peers may have inhibited open discussion about the experiences and beliefs of participants when reporting their own actions. It is possible that a more honest account of personal experience may have been recorded if individual interviews had been employed.

Additionally, the collection of observational data alongside focus group data would have enhanced the rigour of the current findings by providing real-time observations of when and where maternal observations were completed or not completed. Future research could incorporate observation as a method to enhance data triangulation. Further research could include replication of this study in other geographical areas of the UK to gain a wider understanding of inter-Trust differences. Alternatively, a similar study could be conducted to explore this issue from the perspective of MSWs, medical staff or of women accessing maternity care.

## Conclusions

This paper contributes to the literature through its focus on midwives’ experiences of performing maternal observations in England. The findings offer new insights on the reasons for non-completion, including the delegation of tasks to MSWs, completion being more likely in areas with high staffing ratios, the priority placed on these observations, and response of more senior clinicians to escalation. Future training should ensure that both MSWs and midwifery staff are appropriately trained and that there is clarity regarding the responsibilities of midwifery staff and MSWs with regard to clinical observations, emphasising the importance of performing maternal observations and ensuring the effective operation of agreed escalation protocols. In addition, reconfiguring care teams to include more ‘routine’ interaction between midwives and medical staff would be beneficial. The clear message from these findings is that action is required in the areas identified in this study to improve completion rates of maternal observations. Without such action more women are likely to experience avoidable morbidity and mortality, as noted in a succession of CEMD reports.

## Additional files

Additional file 1: Topic Guide for Midwives. Topic Guide for Midwives. Topic Guide used during focus groups with midwives. (DOCX 18 kb)

Additional file 2: Topic Guide for Supervisors of midwives. Topic Guide for Supervisors of midwives. Topic Guide used during focus groups with supervisors of midwives. (DOCX 18 kb)

## Abbreviations

CEMD: Confidential Enquiry into Maternal Deaths
EWS: Early Warning System
LSA: Local Supervising Authority
MEWS: Modified Early Warning Scoring System
MSW: Midwifery Support Worker
NICE: National Institute for Health and Care Excellence
NMC: Nursing and Midwifery Council
RCOG: Royal College of Obstetricians and Gynaecologists
SoM: Supervisor of Midwives
