# Supplementary material for: Midwives’ experiences of performing maternal observations and escalating concerns: a focus group study
Source: BMC Pregnancy Childbirth. 2017 Sep 2;17:282. doi: 10.1186/s12884-017-1472-8 (PMC5581429; doi:10.1186/s12884-017-1472-8)
Supplement: Supplementary file 1 — Topic Guide for Midwives. Topic Guide for Midwives. Topic Guide used during focus groups with midwives. (DOCX 18 kb) [file 12884_2017_1472_MOESM1_ESM.docx]

## Additional File 1 - Topic Guide for Midwives

**Midwives’ views of the facilitators and barriers to performing maternal observations**

**FOCUS GROUP TOPIC GUIDE - MIDWIVES**

Facilitator’s welcome, introduction and instructions to participants

Welcome and thank you for volunteering to take part in this focus group. You have been asked to participate as your point of view is important. I realise that you are busy and I appreciate your time.

**Introduction**: The purpose of this focus group is to explore your thoughts and hear your views as we explore what is the importance of performing maternal observations in everyday midwifery practice? If barriers and facilitators are identified we would also like to hear your thoughts on how these can be supported or addressed in clinical practice.

The focus group discussion will take no longer than 1 hour. I will record the discussion and once transcribed the data will be anonymised so quotes from the data can be used in reports but participants will not be identifiable. If you wish to withdraw from the study you can do so up to the completion of the focus group. Once the data collected from the focus group is transcribed and anonymised it is not possible to extract your anonymised data.

**Anonymity**: Although the discussion will be audio recorded, I would like to assure you that the data from the discussion will be anonymous. The audio files will be stored safely on a password protected, encrypted USB drive until they are transcribed word for word, once checked, they will be destroyed. The transcribed notes of the focus group will contain no information that would allow individual participants to be linked to specific statements. You should try to answer and comment about your experiences of completing observations in everyday practice as accurately and truthfully as possible. I and the other focus group participants would appreciate it if you would refrain from discussing the comments of other group members outside the focus group. If there are any questions or discussions that you do not wish to answer or participate in, you do not have to do so; however, please try to answer and be as involved as possible.

**Ground rules**

• The most important rule is that only one person speaks at a time. There may be a temptation to jump in when someone is talking but please wait until they have finished.

• There is no right or wrong answer

• You do not have to speak in any particular order

• When you do have something to say, please do so by raising your hand. There are many of you in the group and it is important that I obtain the views of each of you

• You do not have to agree with the views of other people in the group

• Does anyone have any questions?

• OK, let’s begin

**Warm up** – ice breaker

• First, I’d like everyone to introduce themselves. Can you tell us your name and your area of clinical practice?

**Introductory question**

I am just going to give you a couple of minutes to think about your own clinical experiences and consider completing observations in everyday practice. Please remember we are also keen to explore some potential solutions to support the completion of maternal observations in everyday practice. Is anyone happy to share their experience?

**Additional questions**

- What are your thoughts about completing maternal observations in everyday practice?

Why are observations performed?

How important do you think performing observations is?

Is this something you do every day?

Is it easy/difficult?

- What are your thoughts about all women having routine observations whilst they are in hospital?

Are women patients?

Do ‘normal’ women need observations performing?

How often?

How important is this task? Are other tasks of higher priority?

- What are your thoughts about the current national guidance in terms of the frequency intervals that are suggested for observations after CS, in the antenatal and postnatal period and in labour?

NICE guidance? Local guidance?

- What are your thoughts about the equipment that is used for completing observations? How easy is it to use? Do you have equipment available?

What sort of equipment do you use and why?

- What are your thoughts about your training and education with regards to maternal observations? If positive, why? If negative, why? (Simulation/lectures/physiology/pre/post registration)
- Who do you think should carry out maternal observations and can you explain why you have identified this group of staff? If MW-why?

If MSW are identified- Can you tell me what your experiences of working alongside MSWs completing observations are? If positive, why? If negative, why?

How much training have the MSW had to complete this task?

How do you feel about MSWs completing maternal observations?

- What are your experiences of referring women for urgent medical review-this can be in the community or within the hospital?

What barriers have you experienced? (GP, Triage A&E, Inpatient).

How does this make you feel?

How do you feel about escalating concerns to senior clinicians?

- What do you think are some of the solutions to the issues that you have raised today?

Provide a summary of issues as prompts if required.

**Concluding question**

• Of all the things we’ve discussed today, what would you say are the main facilitator/barrier to completing observations and what can we do to support practice?

**Conclusion**

• Thank you for participating.

• Your opinions will be a valuable asset to the study

• We hope you have found the discussion interesting

• If there is anything you are unhappy with or wish to complain about, please speak to me later

• I would like to remind you that any comments featuring in this report will be anonymous

- Remember I will be sending you a draft copy of the questionnaires prior to sending out to all the midwives. Your comments would be greatly appreciated.
- Thank you
